# Luteinizing hormone supplementation in women with hypogonadotropic hypogonadism seeking fertility care: Insights from a narrative review

**DOI:** 10.3389/fendo.2022.907249

**Published:** 2022-08-01

**Authors:** Noemi Di Segni, Andrea Busnelli, Matteo Secchi, Federico Cirillo, Paolo Emanuele Levi-Setti

**Affiliations:** ^1^ Division of Gynecology and Reproductive Medicine, Department of Gynecology, Fertility Center, IRCCS Humanitas Research Hospital, Milan, Italy; ^2^ Department of Biomedical Sciences, Humanitas University, Milan, Italy

**Keywords:** LH, LH supplementation, ART, hypogonadotropic hypogonadism, infertility

## Abstract

The management of infertile women affected by hypogonadotropic hypogonadism (HH) or conditions mimicking it is particularly challenging. In the present narrative review, we aimed to synthesize the available evidence on the benefit (if any) of exogenous luteinizing hormone (LH) supplementation in this group of patients. Available data support LH supplementation in women with organic or functional HH. On the contrary, the benefit of exogenous LH on reproductive outcomes both in advanced maternal age patients and in cases of depletion of FSH and LH levels induced by GnRH analogues has not been demonstrated. unfortunately, the inhomogeneous study populations as well as the methodological heterogeneity between studies focused on women affected by conditions mimicking HH do not allow reliable conclusions to be drawn.

## Introduction

According to the International Committee for Monitoring Assisted Reproductive Technologies (ICMART), hypogonadotropic hypogonadism (HH) is defined as “gonadal failure associated with reduced gametogenesis and reduced gonadal steroid production due to reduced gonadotropin production or action” (1). The possible causes of HH are reported in **Table 1** (2–4). The ICMART definition follows the traditional concept but, at the same time, broadens its boundaries by including an exclusively functional etiopathogenesis (2).

**Table 1:** Causes of hypogonadotropic hypogonadism in women.

**Etiology**	
A. Congenital HH a.1 Kallmann Syndrome, Prader Willy Syndrome or other genetic mutation a.2 Idiopathic hypogonadotropic hypogonadism B. Acquired HH b.1 ORGANIC b.1.1. Infiltrative or infectious pituitary lesions (e.g., tumor, hemochromatosis, sarcoidosis, histiocytosis X, thalassemia, granuloma, abscess) b.2 FUNCTIONAL b.2.1 Inadequate caloric intake and assimilation (e.g., eating disorders, malabsorption) b.2.2. Excessive caloric expenditure (e.g., excessive exercise) b.2.3 Stress (e.g., environmental stressors, certain personality traits and psychological disorders) b.2.4 Hypermetabolic states (e.g., severe infections, burns, traumatic brain injury, organ transplant and hyperthyroidism) b.2.5 Age-related impairment of GnRH pulses b.3 IATROGENIC b.3.1 GnRH analogues b.3.2. Drugs (e.g., sex steroids) b.3.3 Pituitary irradiation, trauma or surgery b.3.4 Opiates or alcohol abuse

Women affected by the conditions listed in **Table 1** are, in the vast majority of cases, infertile and, therefore, they frequently refer to an Infertility Unit to receive a diagnosis and a proper treatment. Women selected for assisted reproductive technology (ART) are at increased risk of conditions mimicking HH for at least three other reasons: i) in most cases, they approach treatment at an advanced stage of their reproductive life span; ii) ovarian stimulation (OS) protocols include gonadotropin releasing hormone (GnRH) analogues; iii) a low affinity of luteinizing hormone (LH) and follicle stimulating hormone (FSH) to their receptors are often unrecognized till an unexpected low response to OS for ART (5–8).

The management of patients affected by organic, functional or iatrogenic HH seeking fertility care is particularly challenging and several therapeutic strategies have been proposed.

In the present narrative review, we focused on exogenous LH supplementation. LH exerts two crucial activities during folliculogenesis. First of all, it induces androgen production in theca cells. Second, during the intermediate follicular phase, it cooperates with FSH in stimulating the local production of inhibin B and growth factors. Among these, insulin like growth factors 1 and 2, which are expressed in both granulosa and theca cells, are of utmost importance in promoting follicular maturation (9–11). Based on the notions learned from physiology, the administration of LH combined with FSH during OS in LH deficient women was hypothesized to have beneficial effects on growing follicles and, as a result, on the fertility treatments success rate (9, 10). Furthermore, the addition of exogenous LH might benefit the endometrium by decreasing the risk of a premature progesterone rise (12).

Herein, we aimed at evaluating the effect (if any) of exogenous LH supplementation on fertility related outcomes in women suffering from HH.

## Materials and methods

The present narrative review was restricted to published research articles that reported data relevant to the effect of LH supplementation in women affected by HH or conditions mimicking HH,on fertility treatments outcomes. We searched MEDLINE, Embase and Scopus, from database inception to 1 May 2022. Searches were limited to studies in humans and were conducted using the following terms: ‘hypogonadotropic hypogonadism’ and ‘luteinizing hormone supplementation’ OR ‘luteinizing hormone supplementation’ and ‘ovulation induction’ OR ‘luteinizing hormone’ and ‘*in vitro* fertilization’ OR ‘luteinizing hormone’ and ‘intracitoplasmic sperm injection’ OR ‘luteinizing hormone’ and ‘ advanced maternal age’ OR ‘luteinizing hormone’ and ‘GnRH analogue’ OR ‘luteinizing hormone supplementation’ and ‘gonadotropin’s receptor’.

## Women with depleted basal FSH and LH serum levels

Organic functional HH (World Health Organization (WHO) group I anovulation) determines anovulation, amenorrhea and subsequent infertility (13). Considering the many possible causes, the clinical and hormonal profile of women affected by HH can be very heterogeneous. It has been assumed that, in patients with very low gonadotropins level, in addition to the stimulation with r-FSH a minimum threshold of serum LH is necessary to promote meiosis and final stages of antral follicular growth (14). In particular, the presence of an “LH therapeutic window” was hypothesized. According to this theory, the patients who benefit most from LH supplementation would be those with a mean baseline LH level equal to 1.0 IU/L. On the contrary, in women with LH levels higher than 1.7 IU/L, LH supplementation was deemed to be ineffective (15).

In affected women with an intact pituitary function, pulsatile GnRH therapy can be used to restore the periodic release of FSH and LH, resulting in ovulation. However, effective use of GnRH requires frequent administration (every 60–120 min) and the use of a portable pump injecting the drug either intravenous (iv) or subcutaneous (sc) for several weeks. The alternative therapeutic option is the administration of: i) human menopausal gonadotropin (hMG) (which contains both FSH and LH), ii) a combination of recombinant (r)FSH and recombinant (r)LH, iii) low doses of human chorionic gonadotropin (hCG) (16).

The first randomized clinical trial (RCT) designed to test the efficacy of rLH in HH women was conducted by the European Recombinant Human LH Study Group (16). Patients were randomly assigned to receive 0, 25, 75, or 225 IU rLH once daily in addition to 150 IU rFSH once daily for up to 20 days. Authors demonstrated that, in a dose-related manner, rLH promoted estradiol (E2) secretion, enhanced the effect of FSH on follicular growth, and permitted successful luteinization of follicles when exposed to hCG. In particular, patients who received 75 or 225 IU rLH were more sensitive to FSH than patients who received 25 IU or no rLH. Furthermore, authors observed that when FSH is administered alone to stimulate follicular development, E2 secretion is minimal, resulting in deficient endometrial growth. In addition, when exposed to hCG, these follicles frequently fail to luteinize. Importantly, the group that received 225 IU rLH had a smaller number of growing follicles when compared with the group who received 75 IU rLH. This could suggest an LH ceiling effect, whereby some secondary follicles underwent atresia due to their high sensitivity to LH (17). Loumaye et al. confirmed this effect showing that rLH when administered alone can trigger follicular growth arrest (18). The optimal rLH daily dose has been questioned also by a subsequent prospective, randomized, parallel-group, multicenter trial (15). In this small study, authors provided evidence suggestive of an LH threshold: follicular development was suboptimal when less than 75 IU/day rLH was administered (15).

The distribution and terminal half-lives for rLH are approximately a quarter those of rFSH when administered intravenously or given subcutaneously. Considering the differential pharmacokinetic and pharmacodynamic properties of both molecules, one may thus infer that the administration of rLH at narrower and repeated time intervals could be helpful to reduce serum gonadotrophin fluctuations between dose administrations, potentially improving drug accumulation and serum LH steady-state concentration (16). To untangle this issue, Awwad et al. conducted a non-randomized controlled pilot study aimed at investigating whether split daily doses of rLH was more efficacious than the single daily dose in supporting follicular development and ovulation in primary HH (13). Twenty-seven women with HH received a 150 IU fixed daily subcutaneous dose of rFSH, supplemented by 75 IU daily dose of rLH given either as a single dose (n = 9; single-dose group) or as four equally divided doses (n=18; split-dose group). Although lacking statistical significance, the proportion of women in the rLH split-dose group who fulfilled all three end points (i.e., at least one follicle ≥ 17 mm in diameter, pre-ovulatory serum E2 ≥ 400 pmol/l and a midluteal progesterone ≥ 25 nmol/l) was higher than the single-dose group (72.2% versus 55.6%). There were no serious untoward side effects. Authors concluded that administering rLH in split daily doses could provide superior results compared with the traditional single daily dose (13). The statistical power of the study is limited and additional evidence would be needed. On the other hand, split dose is not considered ‘patient friendly’ and, not surprisingly, no other researcher has further investigated this issue.

Some years later, Shoham et al. conducted a RCT in 25 medical centers in 4 countries. Patients with HH who desired pregnancy were randomized to receive either 75 IU rLH and 150 IU rFSH, or placebo and 150 IU rFSH. Results showed that 16 out of 24 patients treated with rLH and rFSH achieved follicular development compared with 2 out of 10 patients receiving placebo (*p =* 0.023) (19). Which exogenous source of gonadotropins was the most effective in HH women has also been a matter of debate. Carone et al. compared the efficacy of rFSH/rLH in a 2:1 ratio with highly purified hMG (hMG-HP) urinary extract in women affected by WHO type 1 anovulation. Included patients were randomly assigned to receive either 150 IU hMG-HP (150 IU FSH + 150 IU LH-like activity) (n=18 women) or 150IU rFSH + 75IU rLH daily (n=17 women) for a maximum of 16 days. Following a total of 70 cycles, 70% of rFSH/rhLH treated patients met the primary endpoint (i.e., at least one follicle ≥ 17 mm in diameter, pre-ovulatory serum E2 ≥ 400 pmol/l and a midluteal progesterone ≥ 25 nmol/l) versus 88% in the hMG-HP group (*p*=0.11). However, pregnancy rate in the rFSH/rLH group was 55.6% compared to 23.3% in the hMG-HP group (*p*=0.01) (20). Data published by Carone et al. were also re-analysed in a public health perspective: rFSH + rLH generated an incremental cost effectiveness ratio (ICER) equal to €2,007.30 compared to hMG-HP and the average cost per pregnancy was estimated to be €3,990.00 for recombinant strategy and €5,439.80 for urinary strategy (21).

The few data about the safety profile of both rFSH/rLH combination and hMG are reassuring. Further comparative studies are warranted to investigate the tolerability, acceptability, and other adverse events, such as the risk of ovarian hyperstimulation syndrome of rFSH/rLH compared to the conventional hMG regimens to stimulate HH patients (17).

## LH deficiency induced by GnRH analogue protocols

In the 1980s, the introduction of GnRH analogues revolutionised the efficacy of assisted reproductive techniques (ART). In fact, the so-called ‘downregulation protocols’, thanks to their ability in preventing the endogenous LH surge, reduced the rate of cycle cancellation, improved the ART outcomes and enabled some flexibility in scheduling oocyte retrieval (22). The administration of GnRH antagonist during OS determines a rapid and significant fall in LH levels. Usually, the residual hormone is enough to support steroidogenesis in theca cells, and rFSH is sufficient for OS (2). However, in a minority of patients, the 0.25 mg GnRH antagonist daily dose may determine an excessive decrease in LH concentration or a failure in rapidly restoring it (23). A history of ovulatory disorders and previous *in vitro* fertilization (IVF) antagonist cycle treatment seem to be associated with a higher risk of GnRH antagonist hyper-response (23). The impact of such a profound LH suppression on pregnancy outcomes is still debated (23).

GnRH agonists, after an initial increase in LH and FSH secretion (flare up), induce downregulation of the GnRH receptor. A long GnRH-agonist down regulation is thus responsible for a severe reduction of LH secretion (10). The impact of such a decrease in LH serum levels on reproductive outcomes is still a debated and unsolved issue with some studies demonstrating an association between a profound pituitary suppression and lower pregnancy and live birth rates and others denying it (24–27). Furthermore, the different LH threshold values used among studies to define low LH groups further complicate the interpretation and synthesis of available data (2).

Progestins recently emerged as alternatives to GnRH analogues. In fact, they were shown to strongly inhibit the pulsatile GnRH and LH secretion. Progestins are considered an effective option when a fresh embryo transfer (ET) cannot be performed (i.e., fertility preservation, anticipated hyper responders, preimplantation genetic testing (PGT), oocyte donors, etc.) (28, 29). Even after the administration of progestins, in subgroups of patients an excessive suppression of LH secretion may occur.

Against this background, several authors speculated a beneficial effect of LH supplementation in women treated with GnRH analogues. Results of studies investigating this issue are summarized in the next paragraphs. This hypothesis could also be considered valid in protocols involving the administration of progestins but, to date, no studies have yet tested it.

### GnRH antagonist protocol

Several RCTs have been designed to investigate whether the addition of exogenous LH to a GnRH antagonist stimulation protocol could improve the ovarian response and, consequently, pregnancy rates (30–39). Mochtar et al. pooled their results in a Cochrane meta-analysis and found no clear evidence of a difference between rLH/rFSH and rFSH alone in terms of IVF success rates (9). Alviggi et al., in a more recent systematic review, confirmed the absence of a beneficial effect of combined treatment (10). Data syntheses have been criticized for the heterogeneous characteristics of the included populations. In particular, it has been speculated that older women being more prone to develop LH deficiency after GnRH antagonist could be the only ones to benefit from rLH supplementation. Studies published so far, appear statistically homogeneous but differ in some potentially determinant methodological aspects such as the use of oral contraceptive pill (OCP) the cycle prior to OS and the day of OS from which rLH was started. Bosch et al. administered rLH from the beginning of ovarian stimulation after one complete OCP cycle and demonstrated a significantly higher implantation rate in the study group (40). On the contrary, protocols adding rLH from stimulation day 6 did not demonstrate any improvement in IVF cycle outcomes in women 35 years and older. These findings reinforce the concept that the possible beneficial effect of LH requires that its administration starts concomitantly with FSH to achieve optimal steroidogenesis and a better oocyte competence. This role might be especially needed when an OCP is given in the cycle preceding OS, since it determines a marked reduction of LH serum concentration. Although tempting, this hypothesis needs a robust formal confirmation before it can be considered valid for clinical practice (40, 41).

### GnRH agonist protocol

Six RCTs investigated the role of rLH supplementation in women who underwent pituitary suppression with long GnRH agonist protocols (42–47).

Their results are conflicting and prevent from definitive conclusions. Ferraretti et al., conducted the first RCT in the field and observed that the addition of a small amount of rLH to rFSH was associated with significantly higher chances of embryo implantation and pregnancy (42). The same research group, in a subsequent contribution, tested a new stimulation protocol consisting in a sequential administration of 150 IU rLH for 4 days followed by 400 IUI rFSH after downregulation with GnRH agonist. Interestingly, they observed that LH pretreatment was able to decrease the cancellation rate, to improve the *in vitro* performance, and to significantly increase the live birth rates (38). Matorras et al., on stimulation day 6, randomized women aged 35-39 years to receive r FSH alone rFSH+rLH for the remaining ovarian stimulation period. In the ‘intention to treat’ (ITT) analysis, authors observed a significantly higher implantation and live birth rate in the group of women treated with rFSH+rLH. To note, these findings were not confirmed in the ‘per protocol’ (PP) analysis (40). On the other hand, both Tarlatzis et al. and Musters et al. failed to demonstrate a benefit in terms of IVF outcomes associated with the addition of r LH during the late follicular phase of a long GnRH agonist protocol (41, 43). A still debated aspect in GnRH agonist protocols is which LH source is most effective. Orvieto et al., critically presented the available evidence comparing the effect of the two commercially available LH preparations (hMG versus rFSH/rLH) on OS characteristics and in IVF cycle outcomes (48). Authors analysed the results of 10 studies adopting GnRH agonist protocols (three prospective studies of which two RCTs (49, 50) and one prospective observational study (51) and seven retrospective studies (52–58)). Data synthesis showed a higher number of oocytes retrieved but a lower rate of metaphase II (MII) oocytes and lower ongoing pregnancy and live birth rates in women treated with rLH when compared to women treated with hMG. However, the differences failed to reach statistical significance. The author thus established that no firm conclusions can be drawn in favor of a particular source of preparation containing ‘LH activity’ and that large RCTs are needed to confirm the true effect of the source of LH supplementation on IVF outcome (45). More recently, Kirshenbaum et al., in a cross-sectional study, compared OS outcome of two commercially available preparations with different source of LH bioactivity: rFSH/rLH in a fixed 2:1 ratio (Pergoveris^®^, Merck, Darmstadt, Germany) and HP-hMG, containing urinary FSH and LH activity provided by hCG in a fixed 1:1 ratio (Menopur^®^, Ferring pharmaceuticals). Patients treated with rFSH/rLH yielded significantly higher numbers of mature oocytes and fertilized oocytes, with non-significantly lower pregnancy rate per transfer (15% vs 29%, respectively), compared to those treated with HP-hMG (59).

## Advanced maternal age women

Female ageing is characterized by the progressive increase of fully glycosylated FSH variants with a lower affinity for the FSH receptor when compared with the most common isoforms expressed in younger women (2). At the same time, the LH isoforms become progressively more sialylated and less sulfonated over time (2). The impairment of gonadotropins’ action results in reduced steroidogenesis with negative repercussions on ovarian physiology. It was speculated that this form of age-related functional hypogonadism could be corrected or, at least, mitigated by the exogenous LH administration which is expected to increase the androgenic and estrogenic follicle fluid levels. Alviggi et al., summarized the results of RCTs testing this hypothesis and concluded that rLH exerts a beneficial effect in terms of implantation rate in women aged 36-39 years and has no impact in women ≥ 40 years (10). In a subsequent meta-analysis focused on women aged between 35 and 40 years, the same group of researchers demonstrated a positive association between rFSH/rLH cotreatment and clinical pregnancy rate (OR 1.45, 95%CI, 1.05-2.00, p=0.03) (60). However, the only two RCTs reporting the impact of rLH supplementation on the chances of live birth failed to demonstrate any benefit (OR 1.53, 95%CI, 0.50-4.65, p=0.45) (60). As recognized by the authors themselves, available evidence is insufficient and further data is needed. Future research initiatives should focus on narrower age ranges (i.e., 35-37 and 38-40 years) and more homogeneous populations (60). A limitation of data published so far regarding the impact of LH supplementation in advanced maternal age women is the inclusion of studies with relevant methodological differences. Among these, the main one concerns the type of OS protocol adopted. In fact, it is well known that the timing of onset of hypogonadotropic hypogonadism depends on the type of GnRH analogue used. Researchers interested in this issue should keep these methodological aspects in mind when designing future study protocols.

## Genetic variants in gonadotropins’ receptors: Pharmacological implications

LH acts through LH/HCG receptor (LHCGR). LHCGR is expressed on theca cells and, subsequently, develops on granulosa cells (16). Genetic variants of both LH and its receptor can alter the ovarian response to gonadotropins. Such conditions are usually diagnosed following unexpectedly poor responses to OS. Carriers of a common variant of the LH beta chain (rs1800447) are characterized by a less active form of LH that is not able to adequately support FSH activity during the stimulation of follicles’ growth and, as a consequence, results in a reduced response to OS (2, 61). Interactions between FSH receptor (FSHR) and LHCGR polymorphisms are crucial in determining the response to OS protocols. Alviggi et al. observed that the presence of allele C on both FSHR-min29 (rs1394205) and LHCGR-291 (rs 12470652) was associated with an increased ratio between the cumulative rFSH consumption and the total number of oocytes as well as mature oocytes (relative risk (RR) 5.47; CI 95%, 3.13–7.81, p < 0.001) (62).

Lindgren et al. reported that women homozygous for LHCGR N312 required lower doses of exogenous FSH for adequate ovarian response. Considering the dimerization hypothesis, this could indicate that asparagine (N) is associated with a higher receptor sensitivity (63). They also studied the interaction between receptors’ polymorphisms and showed that women homozygous for serine (S) in both considered polymorphisms (FSHR N680S polymorphism and LHCGR N312S polymorphism) had a 4-fold higher chance of pregnancy compared with women homozygous for N in corresponding codons (63). A subsequent cross-sectional study indirectly confirmed Lindgren’s finding (64). In fact, authors found that women heterozygous (N/S) or homozygous (S/S) for serine showed a higher requirement for rLH compared to those homozygous for asparagine (N/N) during OS. Moreover, in the same study, the pregnancy rate was significantly higher in serine carriers who received rFSH/rLH than in those receiving rFSH alone (64). These data combined with others (65) suggests a probable benefit of administering LH supplementation to women undergoing IVF on the basis of their single-nucleotide polymorphism profile (rs2293275) of LHCGR.

## Discussion

The available evidence supports supplementation with exogenous LH for the treatment of WHO group I anovulatory women seeking pregnancy. The choice of the LH daily dose as well as the FSH : LH dose ratio are of utmost relevance in this group of patients. In fact, on the one hand, both an insufficient LH daily dose and a fixed 1:1 ratio were associated with a suboptimal follicular growth. On the other hand, it is equally important not to exceed the proper daily dose to avoid the so called ‘ceiling effect’. Published data suggest that the ideal LH daily dose should be 75 IU (15, 17, 18). However, these recommendations cannot be applied to all patients and under all treatment circumstances. In fact, a daily LH dose of 25 IU is probably insufficient whereas one of 225 IU is associated with a higher risk of follicular atresia, with little being known about intermediate doses. As for the FSH : LH ratio, much of the literature suggests it should be equal to 2:1. However, also this aspect has not been still completely clarified.

These uncertainties should be the incentive for new investigations aimed at establishing criteria that could be useful for the personalization of treatment in this understudied WHO anovulation group.

The efficacy of supplementation with LH in advanced maternal age patients as well as in cases of depletion of FSH and LH levels induced by GnRH analogues has not been demonstrated. Again, further research efforts are needed. In fact, there is a strong suspicion that the discrepancy between what is suggested by the underlying theory (i.e., that iatrogenic HH could benefit from LH supplementation) and what emerged from previous data (9, 10) may be affected by methodological weaknesses as well as by the inhomogeneity of included populations. Age is probably a confounding factor. Indeed, one can speculate that advanced maternal age women are at increased risk of developing HH after GnRH analogues administration. Studies conducted so far have investigated the effect of LH supplementation in women who were considered prone to develop HH without, however, assessing serum LH levels before starting therapy. In GnRH antagonist protocols, attention should be given to those individuals whose LH level increases during the first half of the follicular phase. In fact, such endogenous hormonal trend during the first half of ovarian stimulation could be associated with a sharp decrease in LH immediately after the first GnRH antagonist injection, lack of LH level recovery 24 h later, and, consequently, need for compensation with exogenous LH (23). The proportion of the population selected for IVF experiencing this “abnormal” LH dynamic is probably not negligible. In fact, Kol et al. estimated that 33% of the patients have increased LH level during the first half of OS and are thus at higher risk of hyper-response to the first GnRH antagonist injection (23). The evaluation of serum LH levels should therefore be the cornerstone of future studies’ design.

Data on gonadotropin receptor genetic variants have yet to be considered preliminary. However, this is a fascinating and promising area of research. If the receptor affinities associated with the different polymorphisms were confirmed on a large scale, a new chapter would open in the study of the personalization of OS therapy (2).

The detailed analysis of the other patient categories in which the efficacy of LH supplementation has been investigated (i.e., women with a hyporesponse to exogenous FSH monotherapy and women classified as poor responders to ovarian stimulation (10)) is beyond the scope of this narrative review. In this context, it is however important to underline that patients with these characteristics should not be included in studies aimed at investigating the benefit of LH supplementation on HH to avoid the interposition of confounding factors. The present review has some limitations that need to be acknowledged. First, even though the search of studies was conducted meticulously, the present review cannot be considered compliant with the official guidelines for systematic reviews. Second, we did not carry out a quantitative synthesis of available data. This also prevents us from providing information regarding statistical heterogeneity between studies. On the other hand, it must be recognized that the amount of data on the subject that could be pooled are still scarse and, therefore, the evidence provided by a possible meta-analysis on the subject would have a very limited reliability.

## Conclusions

Knowledge regarding the efficacy of LH supplementation in HH patients has been accumulating in recent years. For the results to be considered reliable and useful in clinical practice, however, a methodological effort is required. First, it is necessary to focus on women with proven low LH serum levels or with failure in rapidly recovering LH concentrations after GnRH analogues administration. Second, one should control the population as much as possible for confounding factors either by designing randomized trials or by applying stringent inclusion criteria (i.e., narrow age ranges, predicted normal response to OS, homogeneous dose and source of exogenous gonadotropins, etc.). Third, as suggested by Bosch and colleagues (2), studies should include information on endocrinological outcomes. In fact, assessing endocrine parameters such as serum testosterone, serum E2 and the E2/oocyte ratio could help in clarifying, in descriptive studies, the endocrinological profile during OS of women with depleted FSH and LH serum levels and, in intervention studies, the stimulatory effect exerted by LH during OS on steroidogenesis in both theca and granulosa cells.

## Author contributions

Conceptualization, NDS, AB and PL-S; writing—original draft preparation, NDS; writing—review and editing, AB and MC; visualization, FC; supervision, PL-S.; project administration, PL-S. All authors have read and agreed to the published version of the manuscript

## Conflict of interest

The authors declare that the research was conducted in the absence of any commercial or financial relationships that could be construed as a potential conflict of interest.

## Publisher’s note

All claims expressed in this article are solely those of the authors and do not necessarily represent those of their affiliated organizations, or those of the publisher, the editors and the reviewers. Any product that may be evaluated in this article, or claim that may be made by its manufacturer, is not guaranteed or endorsed by the publisher.

## Abbreviations

ART, assisted reproductive technology; CI, Confidence interval; E2, Estradiol; FSH, Follicle stimulating hormone; FSHR, FSH receptor; GnRH, Gonadotropin releasing hormone; hCG, human chorionic gonadotropin; HH, Hypogonadotropic hypogonadism; hMG, human menopausal gonadotropin; hMG-HP, highly purified hMG; ICMART, International Committee for Monitoring Assisted Reproductive Technologies; IVF, *in vitro* fertilization; LH, Luteinizing hormone; LHCGR, LH/HCG receptor; OCP, oral contraceptive pill; OR, odds ratio; OS, Ovarian stimulation; RCT, randomized clinical trial; rFSH, recombinant FSH; rLH, recombinant LH; WHO, World Health Organization.
